# Effect of respiratory suspension on the computation of volume-based early peak filling rate to late peak filling rate ratio

**DOI:** 10.1186/1532-429X-18-S1-O93

**Published:** 2016-01-27

**Authors:** Amol Pednekar, Jiming Zhang, Claudio Arena, Melissa Andrews, Debra Dees, Benjamin Cheong, Raja Muthupillai

**Affiliations:** 1 Philips Healthcare, Houston, TX USA; 2 Radiology, CHI St. Luke's Health, Houston, TX USA

## Background

In the intact circulation, changes in intrathoracic pressure and/or lung volume will simultaneously induce alterations in cardiac volumes, output, and contractility among other alterations [1]. In this study, we evaluate the impact of respiratory suspension on the computation of volume-based early peak filling rate (EPFR) to late peak filling rate (LPFR) ratio using peak velocity-based Doppler echo measured early peak velocity (E) to peak velocity during atrial contraction (A) measured at the tip of the mitral leaflets as the reference.

## Methods

All imaging for this IRB approved prospective study was performed on a 1.5T commercial MR scanner (Achieva, Philips Healthcare) in 27 volunteers (16 m/16 f; age 48(20-66)yrs). *MRI*: Identical imaging parameters were used for breath held (BH) (17 subjs), and free breathing (FB) (10 subjs) cine SSFP sequences (TR/TE/flip angle: 3/1.5/60°); acqd voxel size: 2.25 × 2.25 × 8 mm^3^; SENSE:2, temp res: 10-15 ms; acq time: 18 RR intervals/slice; covering the LV in short-axis orientation. FB pulse sequence is described in [2]. *Echocardiography*: Subjects were transported to ultrasound (Philips Healthcare, IE 33) on the same scanner bed to minimize physiologic variation and E/A ratio was obtained. *Data Analysis*: CMR expert drawn endocardial contour at end diastole was propagated across the cardiac phases by a semi-automated algorithm. Resultant LV contours were manually adjusted by CMR expert if needed. From these contours time-LV volume curve was further analyzed using custom-written software in MATLAB™. The raw LV volume curve was upsampled by a factor of 4, and the derivative of the time-volume curve was estimated using the method described in [3]. We defined the ratio of EPFR to LPFR as the MR equivalent surrogate of velocity based echo index of E over A ratio. Linear regression and Bland-Altman (BA) analysis was performed on the results obtained with MR and echo to obtain slope (m), coefficient of determination (r^2^), bias (mean of difference), and limits of agreement(LA, 1.96* stdev of diff).

## Results

High frame rate cine SSFP sequence during free breathing provides cine MR images with adequate temporal resolution to estimate MR based index (EPFR/LPFR) of diastolic function. Doppler based E/A ratios were in good agreement with EPFR/LPFR for FB (m=1.07, r^2^ = 0.85, bias = 0.18, LA 0.12). Breath held acquisitions correlated well with Doppler based E/A ratio (m=1.82, r^2^ = 0.69, bias = 0.22) however LA was more than 8 times higher than with FB acquisition. The BA analysis showed a slope of 0.66 for the bias.

## Conclusions

The volume based E/A ratio derived from high temporal resolution cine MR correlated well with velocity based E/A ratio from echo. The complex interactions between respiratory and cardiovascular systems have direct impact on the measurement of volume-based EPFR/LPFR. EPFR/LPFR computed using free breathing acquisitions are in very good agreement with E/A from echo.

**Figure 1:**
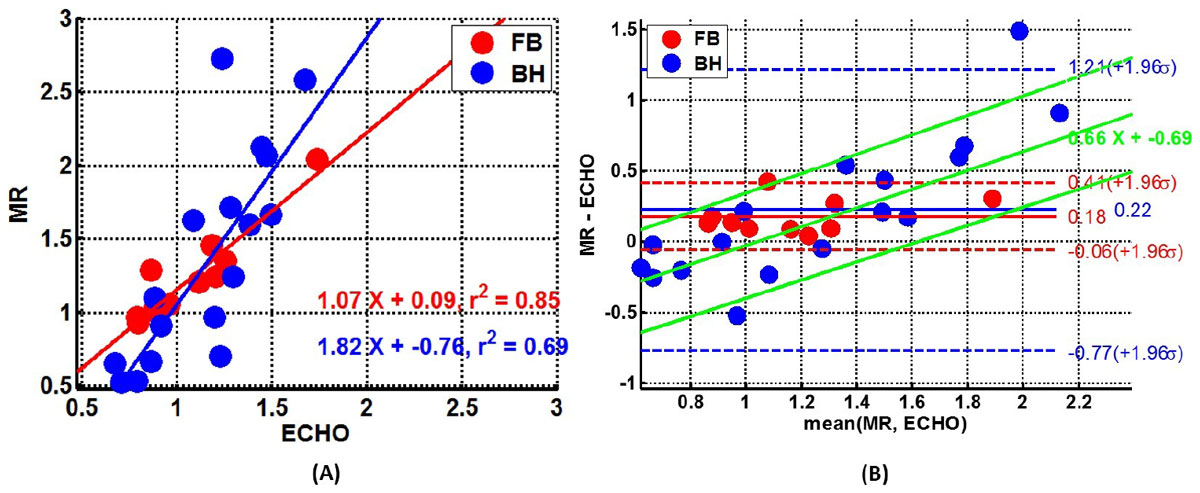
**Linear regression (A) and Bland-Altman (B) for echo (E/A) verses MR (EPFR/LPFR) ratios using breath held (Blue) and free breathing (Red) high temporal resolution cine SFFP sequences**.
